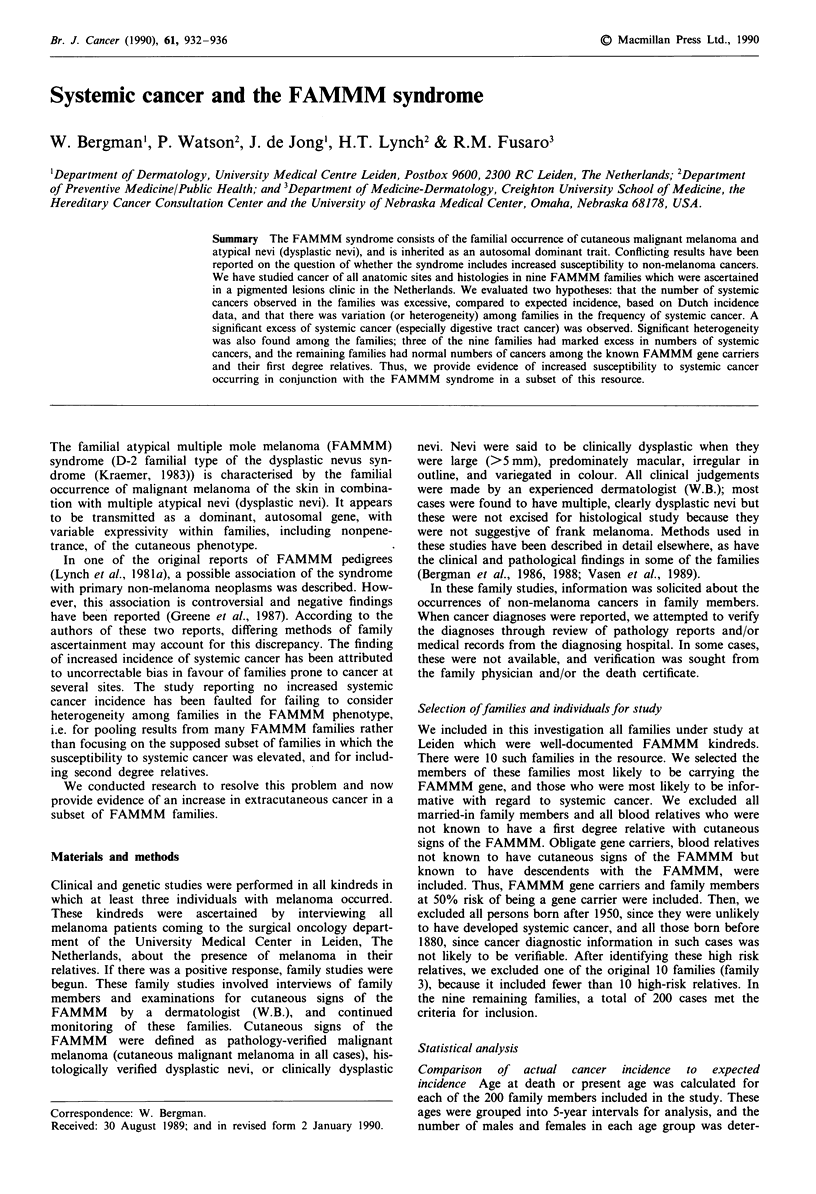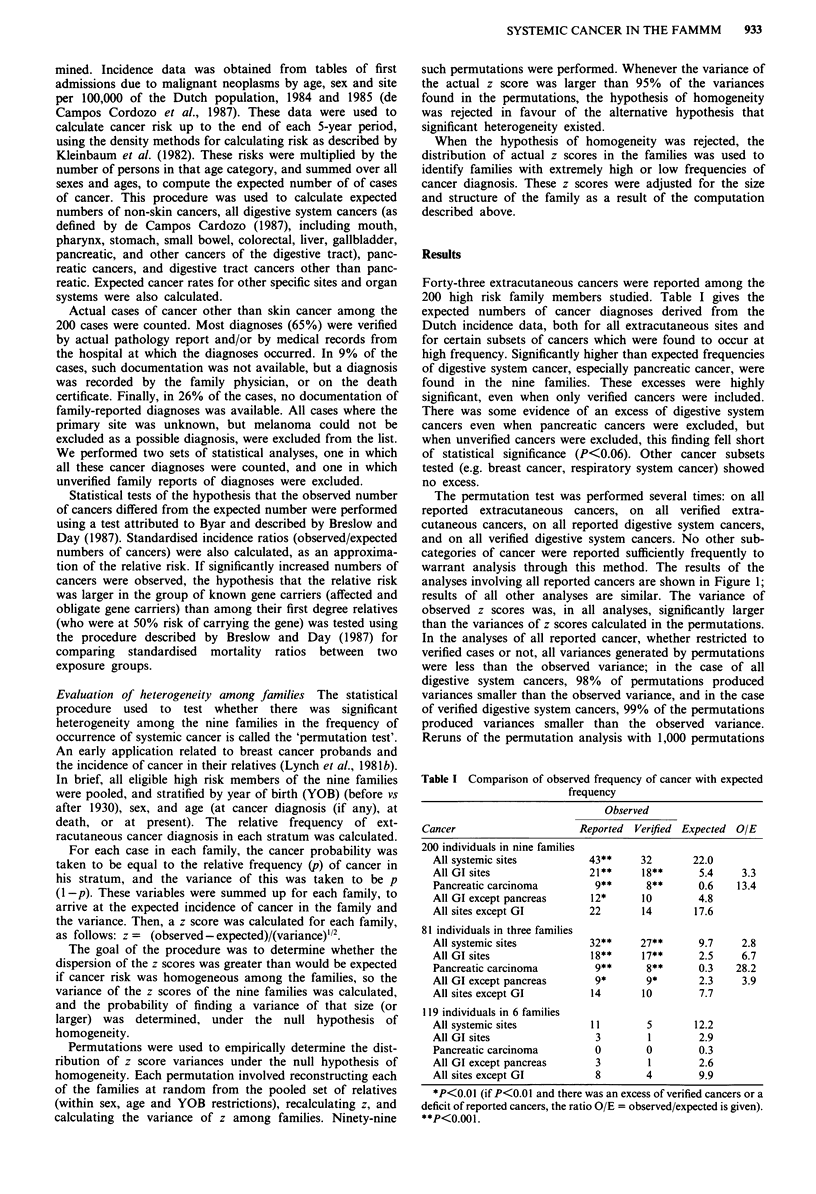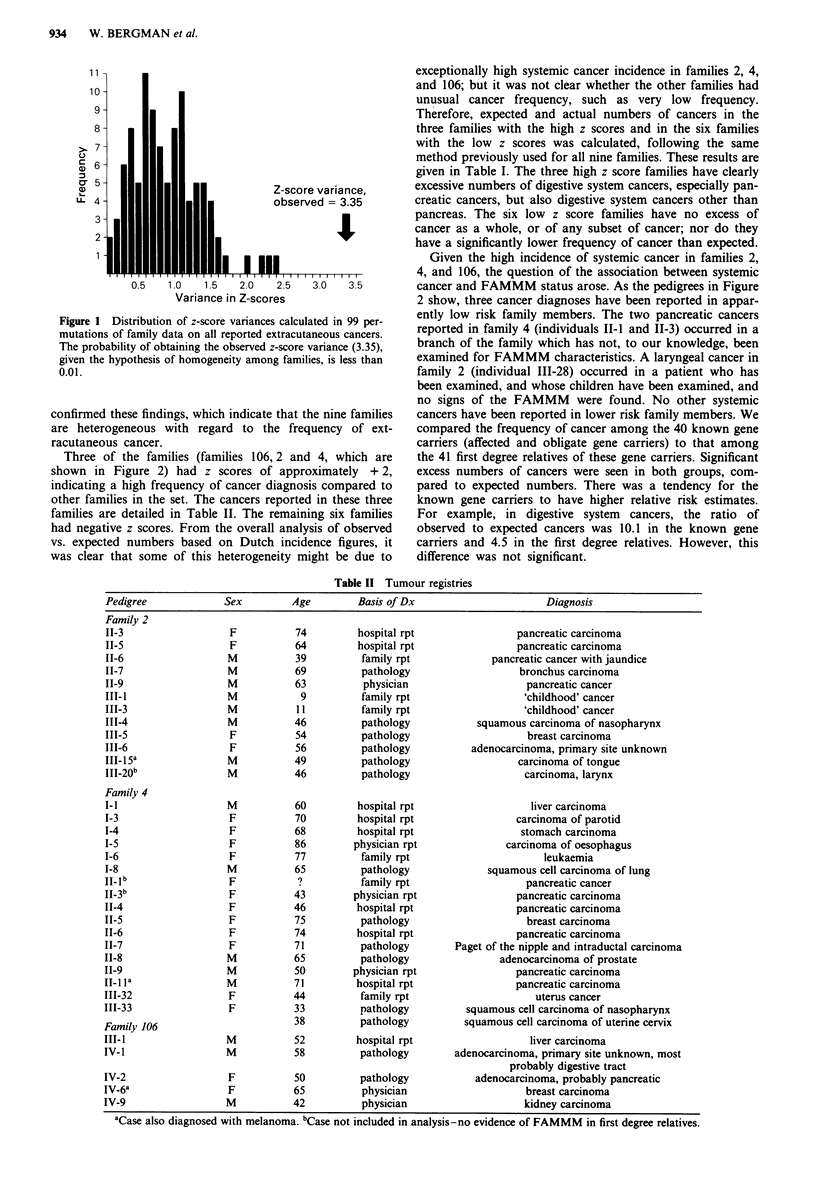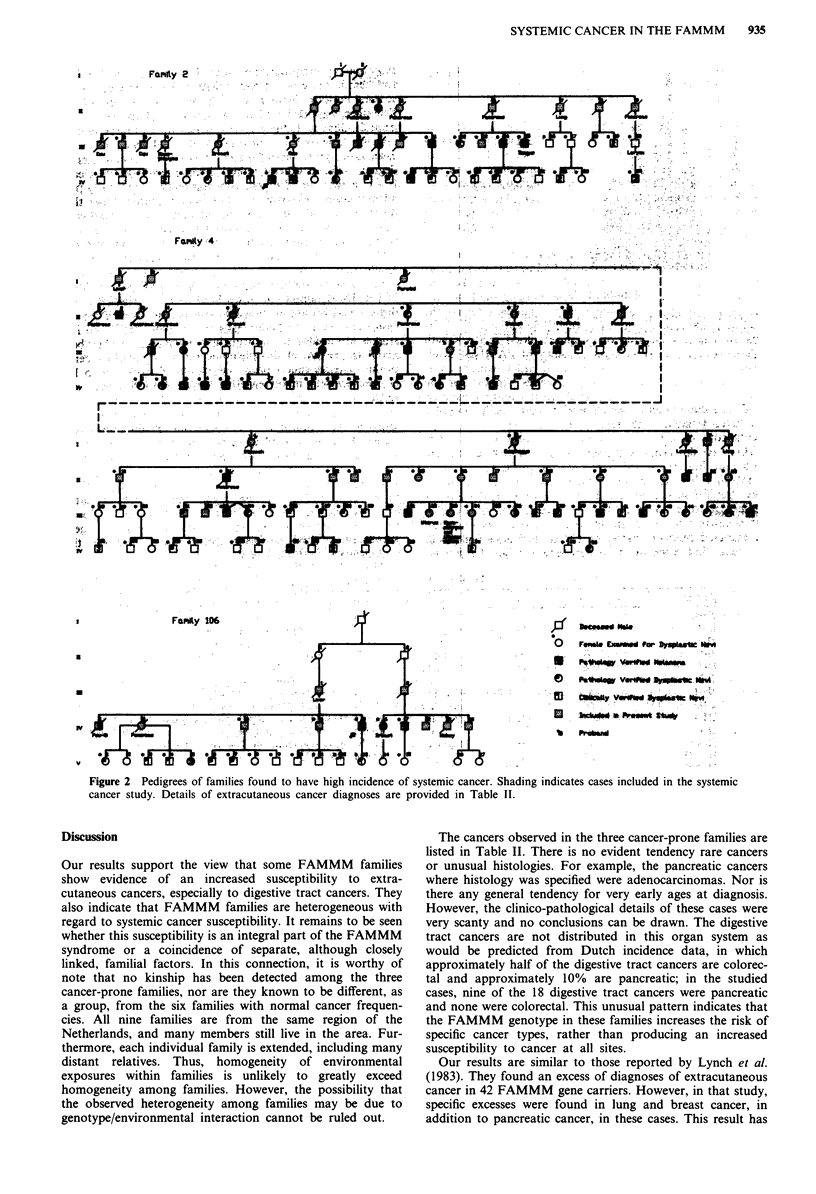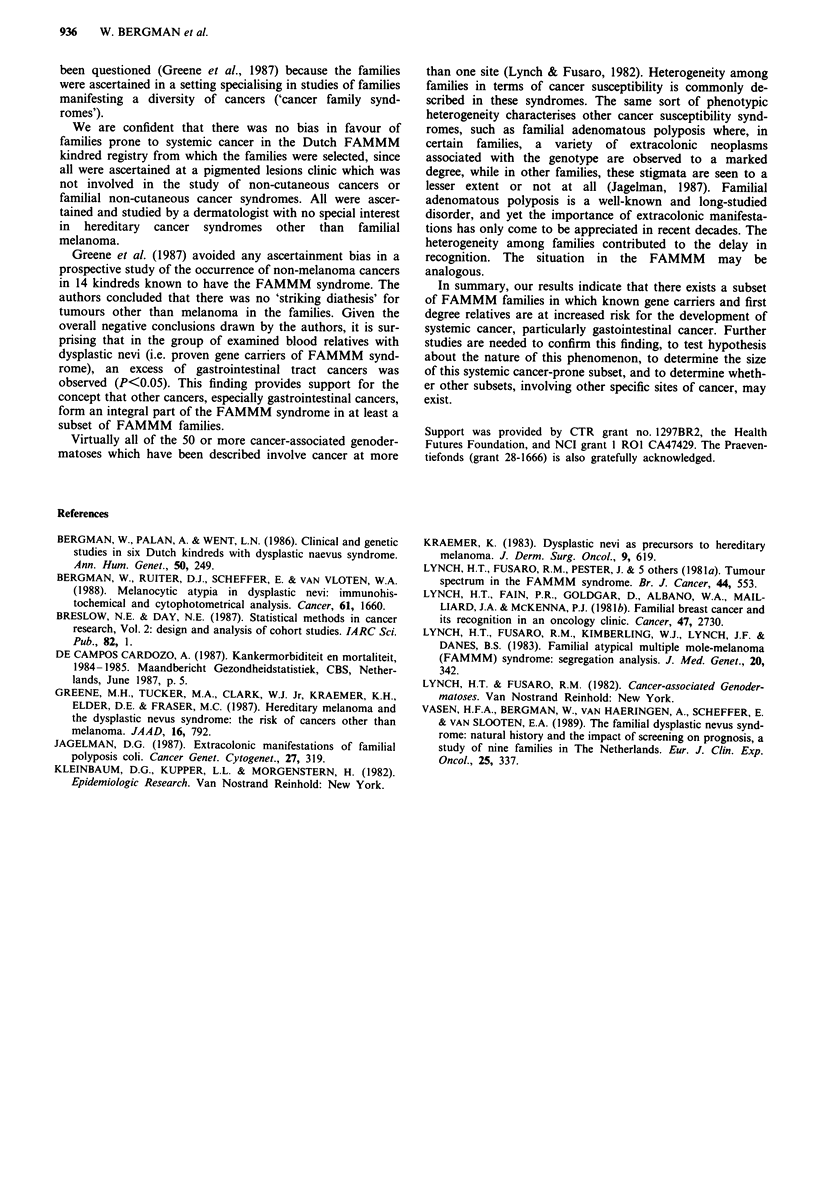# Systemic cancer and the FAMMM syndrome.

**DOI:** 10.1038/bjc.1990.209

**Published:** 1990-06

**Authors:** W. Bergman, P. Watson, J. de Jong, H. T. Lynch, R. M. Fusaro

**Affiliations:** Department of Dermatology, University Medical Centre Leiden, The Netherlands.

## Abstract

The FAMMM syndrome consists of the familial occurrence of cutaneous malignant melanoma and atypical nevi (dysplastic nevi), and is inherited as an autosomal dominant trait. Conflicting results have been reported on the question of whether the syndrome includes increased susceptibility to non-melanoma cancers. We have studied cancer of all anatomic sites and histologies in nine FAMMM families which were ascertained in a pigmented lesions clinic in the Netherlands. We evaluated two hypotheses: that the number of systemic cancers observed in the families was excessive, compared to expected incidence, based on Dutch incidence data, and that there was variation (or heterogeneity) among families in the frequency of systemic cancer. A significant excess of systemic cancer (especially digestive tract cancer) was observed. Significant heterogeneity was also found among the families; three of the nine families had marked excess in numbers of systemic cancers, and the remaining families had normal numbers of cancers among the known FAMMM gene carriers and their first degree relatives. Thus, we provide evidence of increased susceptibility to systemic cancer occurring in conjunction with the FAMMM syndrome in a subset of this resource.


*Br. J. Cancer* (1990), **61**, 932–936 © Macmillan Press Ltd., 1990

### Systemic cancer and the FAMMM syndrome

W. Bergman[1], P. Watson[2], J. de Jong[1], H.T. Lynch[2] & R.M. Fusaro[3]

[1]*Department of Dermatology, University Medical Centre Leiden, Postbox 9600, 2300 RC Leiden, The Netherlands;* [2]*Department of Preventive Medicine/Public Health; and* [3]*Department of Medicine-Dermatology, Creighton University School of Medicine, the Hereditary Cancer Consultation Center and the University of Nebraska Medical Center, Omaha, Nebraska 68178, USA.*

**Summary** The FAMMM syndrome consists of the familial occurrence of cutaneous malignant melanoma and atypical nevi (dysplastic nevi), and is inherited as an autosomal dominant trait. Conflicting results have been reported on the question of whether the syndrome includes increased susceptibility to non-melanoma cancers. We have studied cancer of all anatomic sites and histologies in nine FAMMM families which were ascertained in a pigmented lesions clinic in the Netherlands. We evaluated two hypotheses: that the number of systemic cancers observed in the families was excessive, compared to expected incidence, based on Dutch incidence data, and that there was variation (or heterogeneity) among families in the frequency of systemic cancer. A significant excess of systemic cancer (especially digestive tract cancer) was observed. Significant heterogeneity was also found among the families; three of the nine families had marked excess in numbers of systemic cancers, and the remaining families had normal numbers of cancers among the known FAMMM gene carriers and their first degree relatives. Thus, we provide evidence of increased susceptibility to systemic cancer occurring in conjunction with the FAMMM syndrome in a subset of this resource.

The familial atypical multiple mole melanoma (FAMMM) syndrome (D-2 familial type of the dysplastic nevus syndrome (Kraemer, 1983)) is characterised by the familial occurrence of malignant melanoma of the skin in combination with multiple atypical nevi (dysplastic nevi). It appears to be transmitted as a dominant, autosomal gene, with variable expressivity within families, including nonpenetrance, of the cutaneous phenotype.

In one of the original reports of FAMMM pedigrees (Lynch *et al.*, 1981*a*), a possible association of the syndrome with primary non-melanoma neoplasms was described. However, this association is controversial and negative findings have been reported (Greene *et al.*, 1987). According to the authors of these two reports, differing methods of family ascertainment may account for this discrepancy. The finding of increased incidence of systemic cancer has been attributed to uncorrectable bias in favour of families prone to cancer at several sites. The study reporting no increased systemic cancer incidence has been faulted for failing to consider heterogeneity among families in the FAMMM phenotype, i.e. for pooling results from many FAMMM families rather than focusing on the supposed subset of families in which the susceptibility to systemic cancer was elevated, and for including second degree relatives.

We conducted research to resolve this problem and now provide evidence of an increase in extracutaneous cancer in a subset of FAMMM families.

### Materials and methods

Clinical and genetic studies were performed in all kindreds in which at least three individuals with melanoma occurred. These kindreds were ascertained by interviewing all melanoma patients coming to the surgical oncology department of the University Medical Center in Leiden, The Netherlands, about the presence of melanoma in their relatives. If there was a positive response, family studies were begun. These family studies involved interviews of family members and examinations for cutaneous signs of the FAMMM by a dermatologist (W.B.), and continued monitoring of these families. Cutaneous signs of the FAMMM were defined as pathology-verified malignant melanoma (cutaneous malignant melanoma in all cases), histologically verified dysplastic nevi, or clinically dysplastic nevi. Nevi were said to be clinically dysplastic when they were large (>5 mm), predominately macular, irregular in outline, and variegated in colour. All clinical judgements were made by an experienced dermatologist (W.B.); most cases were found to have multiple, clearly dysplastic nevi but these were not excised for histological study because they were not suggestive of frank melanoma. Methods used in these studies have been described in detail elsewhere, as have the clinical and pathological findings in some of the families (Bergman *et al.*, 1986, 1988; Vasen *et al.*, 1989).

In these family studies, information was solicited about the occurrences of non-melanoma cancers in family members. When cancer diagnoses were reported, we attempted to verify the diagnoses through review of pathology reports and/or medical records from the diagnosing hospital. In some cases, these were not available, and verification was sought from the family physician and/or the death certificate.

### *Selection of families and individuals for study*

We included in this investigation all families under study at Leiden which were well-documented FAMMM kindreds. There were 10 such families in the resource. We selected the members of these families most likely to be carrying the FAMMM gene, and those who were most likely to be informative with regard to systemic cancer. We excluded all married-in family members and all blood relatives who were not known to have a first degree relative with cutaneous signs of the FAMMM. Obligate gene carriers, blood relatives not known to have cutaneous signs of the FAMMM but known to have descendents with the FAMMM, were included. Thus, FAMMM gene carriers and family members at 50% risk of being a gene carrier were included. Then, we excluded all persons born after 1950, since they were unlikely to have developed systemic cancer, and all those born before 1880, since cancer diagnostic information in such cases was not likely to be verifiable. After identifying these high risk relatives, we excluded one of the original 10 families (family 3), because it included fewer than 10 high-risk relatives. In the nine remaining families, a total of 200 cases met the criteria for inclusion.

### *Statistical analysis*

*Comparison of actual cancer incidence to expected incidence* Age at death or present age was calculated for each of the 200 family members included in the study. These ages were grouped into 5-year intervals for analysis, and the number of males and females in each age group was deter-

Correspondence: W. Bergman.
Received: 30 August 1989; and in revised form 2 January 1990.

mined. Incidence data was obtained from tables of first admissions due to malignant neoplasms by age, sex and site per 100,000 of the Dutch population, 1984 and 1985 (de Campos Cordozo *et al.*, 1987). These data were used to calculate cancer risk up to the end of each 5-year period, using the density methods for calculating risk as described by Kleinbaum *et al.* (1982). These risks were multiplied by the number of persons in that age category, and summed over all sexes and ages, to compute the expected number of of cases of cancer. This procedure was used to calculate expected numbers of non-skin cancers, all digestive system cancers (as defined by de Campos Cardozo (1987), including mouth, pharynx, stomach, small bowel, colorectal, liver, gallbladder, pancreatic, and other cancers of the digestive tract), pancreatic cancers, and digestive tract cancers other than pancreatic. Expected cancer rates for other specific sites and organ systems were also calculated.

Actual cases of cancer other than skin cancer among the 200 cases were counted. Most diagnoses (65%) were verified by actual pathology report and/or by medical records from the hospital at which the diagnoses occurred. In 9% of the cases, such documentation was not available, but a diagnosis was recorded by the family physician, or on the death certificate. Finally, in 26% of the cases, no documentation of family-reported diagnoses was available. All cases where the primary site was unknown, but melanoma could not be excluded as a possible diagnosis, were excluded from the list. We performed two sets of statistical analyses, one in which all these cancer diagnoses were counted, and one in which unverified family reports of diagnoses were excluded.

Statistical tests of the hypothesis that the observed number of cancers differed from the expected number were performed using a test attributed to Byar and described by Breslow and Day (1987). Standardised incidence ratios (observed/expected numbers of cancers) were also calculated, as an approximation of the relative risk. If significantly increased numbers of cancers were observed, the hypothesis that the relative risk was larger in the group of known gene carriers (affected and obligate gene carriers) than among their first degree relatives (who were at 50% risk of carrying the gene) was tested using the procedure described by Breslow and Day (1987) for comparing standardised mortality ratios between two exposure groups.

*Evaluation of heterogeneity among families* The statistical procedure used to test whether there was significant heterogeneity among the nine families in the frequency of occurrence of systemic cancer is called the 'permutation test'. An early application related to breast cancer probands and the incidence of cancer in their relatives (Lynch *et al.*, 1981*b*). In brief, all eligible high risk members of the nine families were pooled, and stratified by year of birth (YOB) (before *vs* after 1930), sex, and age (at cancer diagnosis (if any), at death, or at present). The relative frequency of extracutaneous cancer diagnosis in each stratum was calculated.

For each case in each family, the cancer probability was taken to be equal to the relative frequency ($p$) of cancer in his stratum, and the variance of this was taken to be $p(1-p)$. These variables were summed up for each family, to arrive at the expected incidence of cancer in the family and the variance. Then, a $z$ score was calculated for each family, as follows: $z = (\text{observed} - \text{expected})/(\text{variance})^{1/2}$.

The goal of the procedure was to determine whether the dispersion of the $z$ scores was greater than would be expected if cancer risk was homogeneous among the families, so the variance of the $z$ scores of the nine families was calculated, and the probability of finding a variance of that size (or larger) was determined, under the null hypothesis of homogeneity.

Permutations were used to empirically determine the distribution of $z$ score variances under the null hypothesis of homogeneity. Each permutation involved reconstructing each of the families at random from the pooled set of relatives (within sex, age and YOB restrictions), recalculating $z$, and calculating the variance of $z$ among families. Ninety-nine such permutations were performed. Whenever the variance of the actual $z$ score was larger than 95% of the variances found in the permutations, the hypothesis of homogeneity was rejected in favour of the alternative hypothesis that significant heterogeneity existed.

When the hypothesis of homogeneity was rejected, the distribution of actual $z$ scores in the families was used to identify families with extremely high or low frequencies of cancer diagnosis. These $z$ scores were adjusted for the size and structure of the family as a result of the computation described above.

### Results

Forty-three extracutaneous cancers were reported among the 200 high risk family members studied. Table I gives the expected numbers of cancer diagnoses derived from the Dutch incidence data, both for all extracutaneous sites and for certain subsets of cancers which were found to occur at high frequency. Significantly higher than expected frequencies of digestive system cancer, especially pancreatic cancer, were found in the nine families. These excesses were highly significant, even when only verified cancers were included. There was some evidence of an excess of digestive system cancers even when pancreatic cancers were excluded, but when unverified cancers were excluded, this finding fell short of statistical significance ($P<0.06$). Other cancer subsets tested (e.g. breast cancer, respiratory system cancer) showed no excess.

The permutation test was performed several times: on all reported extracutaneous cancers, on all verified extracutaneous cancers, on all reported digestive system cancers, and on all verified digestive system cancers. No other subcategories of cancer were reported sufficiently frequently to warrant analysis through this method. The results of the analyses involving all reported cancers are shown in Figure 1; results of all other analyses are similar. The variance of observed $z$ scores was, in all analyses, significantly larger than the variances of $z$ scores calculated in the permutations. In the analyses of all reported cancer, whether restricted to verified cases or not, all variances generated by permutations were less than the observed variance; in the case of all digestive system cancers, 98% of permutations produced variances smaller than the observed variance, and in the case of verified digestive system cancers, 99% of the permutations produced variances smaller than the observed variance. Reruns of the permutation analysis with 1,000 permutations

**Table I** Comparison of observed frequency of cancer with expected frequency

| Cancer | *Observed* Reported | Verified | *Expected* | *O/E* |
|---|---|---|---|---|
| 200 individuals in nine families | | | | |
| All systemic sites | 43** | 32 | 22.0 | |
| All GI sites | 21** | 18** | 5.4 | 3.3 |
| Pancreatic carcinoma | 9** | 8** | 0.6 | 13.4 |
| All GI except pancreas | 12* | 10 | 4.8 | |
| All sites except GI | 22 | 14 | 17.6 | |
| 81 individuals in three families | | | | |
| All systemic sites | 32** | 27** | 9.7 | 2.8 |
| All GI sites | 18** | 17** | 2.5 | 6.7 |
| Pancreatic carcinoma | 9** | 8** | 0.3 | 28.2 |
| All GI except pancreas | 9* | 9* | 2.3 | 3.9 |
| All sites except GI | 14 | 10 | 7.7 | |
| 119 individuals in 6 families | | | | |
| All systemic sites | 11 | 5 | 12.2 | |
| All GI sites | 3 | 1 | 2.9 | |
| Pancreatic carcinoma | 0 | 0 | 0.3 | |
| All GI except pancreas | 3 | 1 | 2.6 | |
| All sites except GI | 8 | 4 | 9.9 | |

*$P<0.01$ (if $P<0.01$ and there was an excess of verified cancers or a deficit of reported cancers, the ratio O/E = observed/expected is given). **$P<0.001$.

**Figure 1** Distribution of z-score variances calculated in 99 permutations of family data on all reported extracutaneous cancers. The probability of obtaining the observed z-score variance (3.35), given the hypothesis of homogeneity among families, is less than 0.01.

confirmed these findings, which indicate that the nine families are heterogeneous with regard to the frequency of extracutaneous cancer.

Three of the families (families 106, 2 and 4, which are shown in Figure 2) had z scores of approximately +2, indicating a high frequency of cancer diagnosis compared to other families in the set. The cancers reported in these three families are detailed in Table II. The remaining six families had negative z scores. From the overall analysis of observed vs. expected numbers based on Dutch incidence figures, it was clear that some of this heterogeneity might be due to exceptionally high systemic cancer incidence in families 2, 4, and 106; but it was not clear whether the other families had unusual cancer frequency, such as very low frequency. Therefore, expected and actual numbers of cancers in the three families with the high z scores and in the six families with the low z scores was calculated, following the same method previously used for all nine families. These results are given in Table I. The three high z score families have clearly excessive numbers of digestive system cancers, especially pancreatic cancers, but also digestive system cancers other than pancreas. The six low z score families have no excess of cancer as a whole, or of any subset of cancer; nor do they have a significantly lower frequency of cancer than expected.

Given the high incidence of systemic cancer in families 2, 4, and 106, the question of the association between systemic cancer and FAMMM status arose. As the pedigrees in Figure 2 show, three cancer diagnoses have been reported in apparently low risk family members. The two pancreatic cancers reported in family 4 (individuals II-1 and II-3) occurred in a branch of the family which has not, to our knowledge, been examined for FAMMM characteristics. A laryngeal cancer in family 2 (individual III-28) occurred in a patient who has been examined, and whose children have been examined, and no signs of the FAMMM were found. No other systemic cancers have been reported in lower risk family members. We compared the frequency of cancer among the 40 known gene carriers (affected and obligate gene carriers) to that among the 41 first degree relatives of these gene carriers. Significant excess numbers of cancers were seen in both groups, compared to expected numbers. There was a tendency for the known gene carriers to have higher relative risk estimates. For example, in digestive system cancers, the ratio of observed to expected cancers was 10.1 in the known gene carriers and 4.5 in the first degree relatives. However, this difference was not significant.

**Table II** Tumour registries

| *Pedigree* | *Sex* | *Age* | *Basis of Dx* | *Diagnosis* |
|---|---|---|---|---|
| *Family 2* | | | | |
| II-3 | F | 74 | hospital rpt | pancreatic carcinoma |
| II-5 | F | 64 | hospital rpt | pancreatic carcinoma |
| II-6 | M | 39 | family rpt | pancreatic cancer with jaundice |
| II-7 | M | 69 | pathology | bronchus carcinoma |
| II-9 | M | 63 | physician | pancreatic cancer |
| III-1 | M | 9 | family rpt | 'childhood' cancer |
| III-3 | M | 11 | family rpt | 'childhood' cancer |
| III-4 | M | 46 | pathology | squamous carcinoma of nasopharynx |
| III-5 | F | 54 | pathology | breast carcinoma |
| III-6 | F | 56 | pathology | adenocarcinoma, primary site unknown |
| III-15[a] | M | 49 | pathology | carcinoma of tongue |
| III-20[b] | M | 46 | pathology | carcinoma, larynx |
| *Family 4* | | | | |
| I-1 | M | 60 | hospital rpt | liver carcinoma |
| I-3 | F | 70 | hospital rpt | carcinoma of parotid |
| I-4 | F | 68 | hospital rpt | stomach carcinoma |
| I-5 | F | 86 | physician rpt | carcinoma of oesophagus |
| I-6 | F | 77 | family rpt | leukaemia |
| I-8 | M | 65 | pathology | squamous cell carcinoma of lung |
| II-1[b] | F | ? | family rpt | pancreatic cancer |
| II-3[b] | F | 43 | physician rpt | pancreatic carcinoma |
| II-4 | F | 46 | hospital rpt | pancreatic carcinoma |
| II-5 | F | 75 | pathology | breast carcinoma |
| II-6 | F | 74 | hospital rpt | pancreatic carcinoma |
| II-7 | F | 71 | pathology | Paget of the nipple and intraductal carcinoma |
| II-8 | M | 65 | pathology | adenocarcinoma of prostate |
| II-9 | M | 50 | physician rpt | pancreatic carcinoma |
| II-11[a] | M | 71 | hospital rpt | pancreatic carcinoma |
| III-32 | F | 44 | family rpt | uterus cancer |
| III-33 | F | 33 | pathology | squamous cell carcinoma of nasopharynx |
| | | 38 | pathology | squamous cell carcinoma of uterine cervix |
| *Family 106* | | | | |
| III-1 | M | 52 | hospital rpt | liver carcinoma |
| IV-1 | M | 58 | pathology | adenocarcinoma, primary site unknown, most probably digestive tract |
| IV-2 | F | 50 | pathology | adenocarcinoma, probably pancreatic |
| IV-6[a] | F | 65 | physician | breast carcinoma |
| IV-9 | M | 42 | physician | kidney carcinoma |

[a]Case also diagnosed with melanoma. [b]Case not included in analysis–no evidence of FAMMM in first degree relatives.

**Figure 2** Pedigrees of families found to have high incidence of systemic cancer. Shading indicates cases included in the systemic cancer study. Details of extracutaneous cancer diagnoses are provided in Table II.

### Discussion

Our results support the view that some FAMMM families show evidence of an increased susceptibility to extracutaneous cancers, especially to digestive tract cancers. They also indicate that FAMMM families are heterogeneous with regard to systemic cancer susceptibility. It remains to be seen whether this susceptibility is an integral part of the FAMMM syndrome or a coincidence of separate, although closely linked, familial factors. In this connection, it is worthy of note that no kinship has been detected among the three cancer-prone families, nor are they known to be different, as a group, from the six families with normal cancer frequencies. All nine families are from the same region of the Netherlands, and many members still live in the area. Furthermore, each individual family is extended, including many distant relatives. Thus, homogeneity of environmental exposures within families is unlikely to greatly exceed homogeneity among families. However, the possibility that the observed heterogeneity among families may be due to genotype/environmental interaction cannot be ruled out.

The cancers observed in the three cancer-prone families are listed in Table II. There is no evident tendency rare cancers or unusual histologies. For example, the pancreatic cancers where histology was specified were adenocarcinomas. Nor is there any general tendency for very early ages at diagnosis. However, the clinico-pathological details of these cases were very scanty and no conclusions can be drawn. The digestive tract cancers are not distributed in this organ system as would be predicted from Dutch incidence data, in which approximately half of the digestive tract cancers are colorectal and approximately 10% are pancreatic; in the studied cases, nine of the 18 digestive tract cancers were pancreatic and none were colorectal. This unusual pattern indicates that the FAMMM genotype in these families increases the risk of specific cancer types, rather than producing an increased susceptibility to cancer at all sites.

Our results are similar to those reported by Lynch *et al.* (1983). They found an excess of diagnoses of extracutaneous cancer in 42 FAMMM gene carriers. However, in that study, specific excesses were found in lung and breast cancer, in addition to pancreatic cancer, in these cases. This result has been questioned (Greene *et al.*, 1987) because the families were ascertained in a setting specialising in studies of families manifesting a diversity of cancers ('cancer family syndromes').

We are confident that there was no bias in favour of families prone to systemic cancer in the Dutch FAMMM kindred registry from which the families were selected, since all were ascertained at a pigmented lesions clinic which was not involved in the study of non-cutaneous cancers or familial non-cutaneous cancer syndromes. All were ascertained and studied by a dermatologist with no special interest in hereditary cancer syndromes other than familial melanoma.

Greene *et al.* (1987) avoided any ascertainment bias in a prospective study of the occurrence of non-melanoma cancers in 14 kindreds known to have the FAMMM syndrome. The authors concluded that there was no 'striking diathesis' for tumours other than melanoma in the families. Given the overall negative conclusions drawn by the authors, it is surprising that in the group of examined blood relatives with dysplastic nevi (i.e. proven gene carriers of FAMMM syndrome), an excess of gastrointestinal tract cancers was observed ($P<0.05$). This finding provides support for the concept that other cancers, especially gastrointestinal cancers, form an integral part of the FAMMM syndrome in at least a subset of FAMMM families.

Virtually all of the 50 or more cancer-associated genodermatoses which have been described involve cancer at more than one site (Lynch & Fusaro, 1982). Heterogeneity among families in terms of cancer susceptibility is commonly described in these syndromes. The same sort of phenotypic heterogeneity characterises other cancer susceptibility syndromes, such as familial adenomatous polyposis where, in certain families, a variety of extracolonic neoplasms associated with the genotype are observed to a marked degree, while in other families, these stigmata are seen to a lesser extent or not at all (Jagelman, 1987). Familial adenomatous polyposis is a well-known and long-studied disorder, and yet the importance of extracolonic manifestations has only come to be appreciated in recent decades. The heterogeneity among families contributed to the delay in recognition. The situation in the FAMMM may be analogous.

In summary, our results indicate that there exists a subset of FAMMM families in which known gene carriers and first degree relatives are at increased risk for the development of systemic cancer, particularly gastointestinal cancer. Further studies are needed to confirm this finding, to test hypothesis about the nature of this phenomenon, to determine the size of this systemic cancer-prone subset, and to determine whether other subsets, involving other specific sites of cancer, may exist.

Support was provided by CTR grant no. 1297BR2, the Health Futures Foundation, and NCI grant 1 RO1 CA47429. The Praeventiefonds (grant 28-1666) is also gratefully acknowledged.

### References

BERGMAN, W., PALAN, A. & WENT, L.N. (1986). Clinical and genetic studies in six Dutch kindreds with dysplastic naevus syndrome. *Ann. Hum. Genet.*, **50**, 249.

BERGMAN, W., RUITER, D.J., SCHEFFER, E. & VAN VLOTEN, W.A. (1988). Melanocytic atypia in dysplastic nevi: immunohistochemical and cytophotometrical analysis. *Cancer*, **61**, 1660.

BRESLOW, N.E. & DAY, N.E. (1987). Statistical methods in cancer research, Vol. 2: design and analysis of cohort studies. *IARC Sci. Pub.*, **82**, 1.

DE CAMPOS CARDOZO, A. (1987). Kankermorbiditeit en mortaliteit, 1984–1985. Maandbericht Gezondheidstatistiek, CBS, Netherlands, June 1987, p. 5.

GREENE, M.H., TUCKER, M.A., CLARK, W.J. Jr, KRAEMER, K.H., ELDER, D.E. & FRASER, M.C. (1987). Hereditary melanoma and the dysplastic nevus syndrome: the risk of cancers other than melanoma. *JAAD*, **16**, 792.

JAGELMAN, D.G. (1987). Extracolonic manifestations of familial polyposis coli. *Cancer Genet. Cytogenet.*, **27**, 319.

KLEINBAUM, D.G., KUPPER, L.L. & MORGENSTERN, H. (1982). *Epidemiologic Research*. Van Nostrand Reinhold: New York.

KRAEMER, K. (1983). Dysplastic nevi as precursors to hereditary melanoma. *J. Derm. Surg. Oncol.*, **9**, 619.

LYNCH, H.T., FUSARO, R.M., PESTER, J. & 5 others (1981*a*). Tumour spectrum in the FAMMM syndrome. *Br. J. Cancer*, **44**, 553.

LYNCH, H.T., FAIN, P.R., GOLDGAR, D., ALBANO, W.A., MAILLIARD, J.A. & MCKENNA, P.J. (1981*b*). Familial breast cancer and its recognition in an oncology clinic. *Cancer*, **47**, 2730.

LYNCH, H.T., FUSARO, R.M., KIMBERLING, W.J., LYNCH, J.F. & DANES, B.S. (1983). Familial atypical multiple mole-melanoma (FAMMM) syndrome: segregation analysis. *J. Med. Genet.*, **20**, 342.

LYNCH, H.T. & FUSARO, R.M. (1982). *Cancer-associated Genodermatoses*. Van Nostrand Reinhold: New York.

VASEN, H.F.A., BERGMAN, W., VAN HAERINGEN, A., SCHEFFER, E. & VAN SLOOTEN, E.A. (1989). The familial dysplastic nevus syndrome: natural history and the impact of screening on prognosis, a study of nine families in The Netherlands. *Eur. J. Clin. Exp. Oncol.*, **25**, 337.